# Transcriptomic Response of Breast Cancer Cells MDA-MB-231 to Docosahexaenoic Acid: Downregulation of Lipid and Cholesterol Metabolism Genes and Upregulation of Genes of the Pro-Apoptotic ER-Stress Pathway

**DOI:** 10.3390/ijerph17103746

**Published:** 2020-05-25

**Authors:** Benoît Chénais, Marine Cornec, Solenne Dumont, Justine Marchand, Vincent Blanckaert

**Affiliations:** 1 EA2160 Mer Molécules Santé, Le Mans Université, F-72085 Le Mans, France; justine.marchand@univ-lemans.fr (J.M.); vblanck@univ-lemans.fr (V.B.); 2 CHU Nantes, Inserm, CNRS, SFR Santé, Inserm UMS 016, CNRS UMS 3556, Université de Nantes, F-44000 Nantes, France; marine.cornec@univ-nantes.fr (M.C.); solenne.dumont@univ-nantes.fr (S.D.); 3 CRCINA, INSERM, Université d’Angers, Université de Nantes, F-44000 Nantes, France

**Keywords:** apoptosis, breast cancer, cholesterol metabolism, docosahexaenoic acid, ER-stress, migration, invasion, lipid metabolism, unfolded protein response

## Abstract

Despite considerable efforts in prevention and therapy, breast cancer remains a major public health concern worldwide. Numerous studies using breast cancer cell lines have shown the antiproliferative and pro-apoptotic effects of docosahexaenoic acid (DHA). Some studies have also demonstrated the inhibitory effect of DHA on the migration and invasion of breast cancer cells, making DHA a potential anti-metastatic agent. Thus, DHA has shown its potential as a chemotherapeutic adjuvant. However, the molecular mechanisms triggering DHA effects remain unclear, and the aim of this study was to provide a transcriptomic basis for further cellular and molecular investigations. Therefore, MDA-MB-231 cells were treated with 100 µM DHA for 12 h or 24 h before RNA-seq analysis. The results show the great impact of DHA-treatment on the transcriptome, especially after 24 h of treatment. The impact of DHA is particularly visible in genes involved in the cholesterol biosynthesis pathway that is strongly downregulated, and the endoplasmic reticulum (ER)-stress response that is, conversely, upregulated. This ER-stress and unfolded protein response could explain the pro-apoptotic effect of DHA. The expression of genes related to migration and invasion (especially *SERPINE1*, *PLAT*, and *MMP11*) is also impacted by DHA. In conclusion, this transcriptomic analysis supports the antiproliferative, pro-apoptotic and anti-invasive effects of DHA, and provides new avenues for understanding its molecular mechanisms.

## 1. Introduction

Breast cancer is the most frequent malignant tumor in women and accounts for about 25% of all carcinomas diagnosed in women worldwide [1,2]. Despite increasing developments in therapeutic strategies, breast cancer remains a deadly threat to patients [2]; therefore, in addition to efforts for new therapeutic approaches, it is also necessary to promote prevention strategies.

Bioactive molecules from natural sources have increased interest as therapeutics and adjuvants, or for their beneficial dietary effects. Polyunsaturated fatty acids (PUFAs) have demonstrated several, and sometimes controversial benefits for human health [3,4]. Increasing evidence suggests that n-3 PUFA, but not n-6 PUFAs, have anticancer activity and improve the effectiveness of conventional cancer therapy [5,6,7,8,9,10,11,12,13,14]. However, the analysis of PUFAs-enriched diet effects on breast cancer appearance and progression is complex and controversial due to the multitude of cancer typologies and origins and the pleiotropic effect of PUFAs [15,16,17].

Docosahexaenoic acid (DHA, 22:6n-3) is a polyunsaturated fatty acid with its first double bond at the n-3 (or omega-3) position (n-3 PUFA). DHA is synthesized from the essential fatty acid precursor alpha-linolenic acid (18:3n-3), and its main nutritional sources are currently fatty fishes and oily fish. Microalgae such as *Diacronema lutheri* or *Tisochrysis lutea* appear as promising alternatives for DHA supply [11,12,18]. DHA has shown anticancer effects in several types of cancers and, at the cellular level, has demonstrated promising antiproliferative and pro-apoptotic effects in several types of cancers and cell lines [9,19,20,21], among which breast cancer cells [17,22,23]. The mechanisms underlying the anticancer effect of DHA include the regulation of Wnt/β-catenin inhibition, oxidative DNA damage, and mitogen-activated protein kinase activation (reviewed in [18,22,23]). DHA was also shown to induce autophagy whilst suppressing mTOR in human cervical cancer, prostate cancer, lung cancer, and glioblastoma cells [24,25,26,27]. More specifically for breast cancer, several intracellular targets have been identified as being involved in the DHA effect, among which the PKB/akt, and p53 pathways and increased caspase activity (reviewed in [18,23]). Additional mechanisms involved in the pro-apoptotic effect of DHA in breast cancer cells include, but are not limited to, decreased Erk activity [28,29], increased Bax pro-apoptotic enzyme levels or activity and decreased Bcl-XL, increased death receptors (DR-4, TRAIL, and Fas) expression and mitochondrial release of the caspase activator SMAC/Diablo in the MCF-7 cell line [30], PPAR-α overexpression in breast cancer tissue or cells [31,32], increased expression of the stress-induced growth inhibitor 1 (OSGIN1) and transcription factor NFE2L2 in MCF-7 and Hs578T breast cancer cells [33]. However, DHA also increased the activity of antioxidant enzymes (SOD, CAT, and GPX) in breast cancer tissues [31], suggesting that the anti-/pro-oxidant effects of DHA may be dependent on the cell type and concentration used. Finally, DHA also acts as an antiproliferative agent by lengthening the cell cycle at the G2/M transition, such as in the MDA-MB-231 breast cancer cell line [34].

A few studies have shown that diet can affect the metastatic potential of cancer cells known to have a high metastatic phenotype such as breast cancer [35,36]. The anti-invasive effect of DHA, at a concentration ranging from 10 to 100 µM, was highlighted in breast cancer cell lines [31,37,38,39,40,41,42,43]. Moreover, this anti-metastatic effect is corroborated by in vivo animal studies using *fat-1* transgenic mice capable of producing n-3 FA from the n-6 type, leading to abundant n-3 FA with reduced levels of n-6 FA in their organs and tissues, and this was without the need of a dietary n-3 supply [44]. The use of *fat-1* transgenic mice has shown a decrease in tumor growth and the diminution of lung metastasis of syngeneic breast cancer cells in this DHA-rich environment [42].

Therefore, DHA appears as a safe, natural compound that can greatly improve the anticancer properties of anticancer drugs by additive or synergistic interactions [14,45,46]. In addition, n-3 PUFA reduced the risk of obesity-related breast cancer [47] and had protective effects towards the cardiotoxicity of anthracyclines, the most extensively used chemotherapeutics [48,49]. Thus, current results of cohort studies and investigations in cell lines or animal models demonstrated that DHA could reduce tumor cell number by acting as soon as the cell begins its neoplastic transformation through a decrease in proliferation and an increase in apoptosis. However, the cellular targets and mechanisms of action of DHA remain to be further understood, and genome-wide transcriptomic studies are few.

In the present study, MDA-MB-231 cells, which are a commonly used model of triple-negative breast cancer, were submitted to DHA treatment and then analyzed by RNA-sequencing.

## 2. Materials and Methods

### 2.1. Cell Culture and Treatment

The triple-negative breast cancer cell line MDA- MB-231 was purchased from ATCC (Manassas, VA, USA) and routinely grown as monolayers at 37 °C, in a humidified atmosphere with 5% CO_2_, in minimum essential medium (MEM) (Sigma-Aldrich, Saint-Quentin Fallavier, France) supplemented with 10% fetal calf serum (FCS) (Gibco; Invitrogen, Cergy Pontoise, France), 20 mM Hepes, and 2 mM L-glutamine, 100 U/ml penicillin/streptomycin (Sigma-Aldrich).

For subsequent RNA analysis experiments, cells were plated in four biological replicates in six-wells plates for 24 h, then the medium was removed and replaced by fresh medium containing or not 100 µM DHA for 12 h or 24 h. Cells were then dissociated with 0.5 mL of non-enzymatic dissociation solution. After homogenizing, cell viability was determined using the Trypan blue dye exclusion test (Sigma-Aldrich), and counts were performed using a Malassez hemocytometer. The remaining cells were washed with 5 mL sterile phosphate buffer saline (PBS), and centrifuged for 10 min at 200× *g*. The pellet was homogenized, washed again with PBS, and centrifuged under the same conditions. The supernatants were discarded, and cells kept at -80 °C until RNA extraction.

### 2.2. RNA Extraction

Cell pellets (1.6 × 10^6^ cells) were first lysed and homogenized using the QIAshredder kit (Qiagen, Courtaboeuf, France), then total RNAs were extracted using the RNeasy mini kit (Qiagen) according to manufacturer’s instructions. The total RNA was quantified using a Nanodrop^®^ND-1000 spectrophotometer (Thermo Fisher Scientific, Illkirch, France). The quality and integrity of RNA samples were assessed using the 2100 Bioanalyzer and RNA 6000 Nano LabChip kit series II (Agilent Technologies, Les Ulis, France). The RNAs extracted were of good quality, and the RNA integrity number (RIN) was >9 in all cases.

### 2.3. Library Construction and RNA-seq Analysis

For each of the four biological replicates per condition, the library construction was performed from 500 ng of total RNA with SureSelect Strand-Specific RNA Library Prep for Illumina Multiplexed kit (#5190-6410, Agilent Technologies) according to manufacturer’ protocol as previously described [50]. Purifications were carried out with NucleoMag NGS Clean-up and Size Select (#744970.50, Macherey-Nagel, Hoerdt, France). The fragments size of libraries was controlled on D1000 ScreenTape with 2200 TapeStation system (Agilent Technologies). Libraries with P5–P7 adaptors were specifically quantified on the LightCylcer ^®^ 480 Instrument II (Roche Life Science, Meylan, France) and normalized with DNA Standards (1–6) (#KK4903, Kapa Biosystems—CliniSciences, Nanterre, France). Then, 11.5 picomolar of each library was pooled and prepared according to the denaturing and diluting libraries protocol for the Hiseq and GAIIx (part#15050107 v02, Illumina, Paris, France) for cluster generation on the cBotTM system. Paired-end sequencing (2 × 100 cycles) was carried out in four lanes on HiSeq^®^ 2500 system (Illumina) in TruSeq v3 chemistry according to the instructions of HiSeq^®^ 2500 System Guide (part#15035786 v01, Illumina). After demultiplexing and quality control with fastQC_0.11.2 (http://www.bioinformatics.babraham.ac.uk/projects/fastqc/), Illumina adapter were trimmed with Cutadapt-1.2.120 and reads with a Phred quality score below 30 were filtered with prinseq-lite-0.20.321,22. Reads were aligned against the human hg19 reference genome with Tophat2.0.1023 counted with the HTseq-count from HTSeq-0.5.4p524, and differential analysis was performed with DESeq225. Differentially expressed genes were selected when the Log2 fold change ≥1 and *p* < 0.001.

### 2.4. Quantitative RT-PCR

RNA reverse transcription was performed using the GoScript reverse transcriptase (Promega, Madison, WI) with random primers and 1 µg of total RNA. The obtained RT-products were diluted 1/16 for quantitative real-time PCR (qPCR), which was performed in triplicates using the GoTaq^®^qPCR Master Mix (Promega) with CXR reference dye (Promega) and the real-time thermal cycler StepOne Plus (Applied Biosystems). The fold change was calculated as previously described in [51] using Glyceraldehyde-3-Phosphate Dehydrogenase (*GAPDH*) and Actin-β (*ACTB*) as housekeeping genes (N = 3). Primers used for qPCR amplification are displayed in Table S1 and were purchased from Eurofins (Nantes, France).

### 2.5. Statistical Analysis

The RNA-seq analysis was done in four biological replicates, while quantitative RT-PCR was done using three biological replicates. Individual data points are plotted with mean ± SD. Statistical analyses were done using two-way ANOVA-multiple comparisons with difference considered statistically significant at *p* < 0.001 (GraphPad Prism 6, GraphPad Software, San Diego, CA, USA).

## 3. Results and Discussion

### 3.1. Global Overview of DHA Impact on MDA-MB-231 Cells Transcriptome

**Figure 1:**
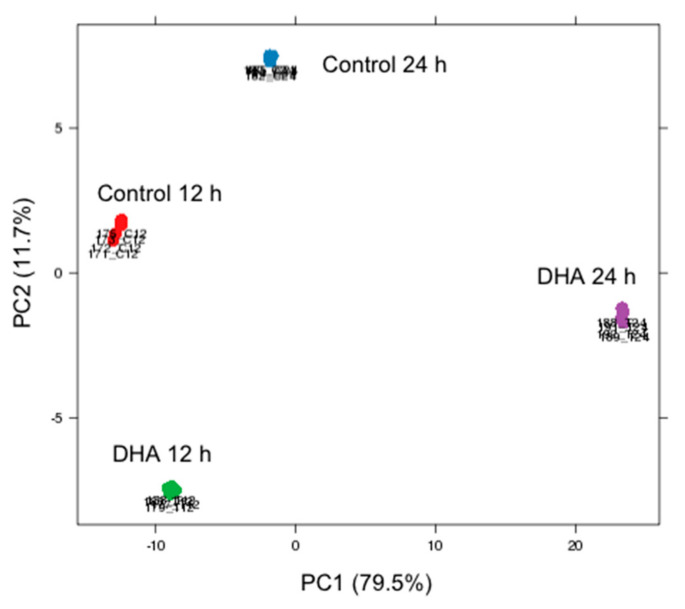
Principal component analysis (PCA) of RNA-seq samples. PCA of samples analyzed in quadruplicate. PC1 (79.5%) shows a very significant difference between the samples treated with DHA for 24 h (violet spots) versus all other samples. PC2 (11.7%) discriminates control samples (red and blue spots) from treated samples (green and violet spots). These two axes explain 91.2% of the data variability, with a very good intra-group homogeneity.

In order to gain further insights into the molecular mechanisms triggered by DHA in breast cancer cells, we have undertaken a transcriptomic analysis of MDA-MB-231 cells treated with 100 µM of DHA, a concentration that has proven its efficiency to reduce proliferation, induce apoptosis, and prevent invasion in this cell line [37,38]. The RNA-seq analyses were performed on biological quadruplicates, and the average depth of coverage obtained after sequencing was 37 million pairs of reads. The principal component analysis (PCA) of the samples shows a very high intra-group homogeneity, since more than 90% of the variability of the data is explained by the first two axes (Figure 1). The samples treated with DHA for 24 h stand out clearly (PC1: 79.5%) from the rest of the samples, which shows that the effect of the treatment appears particularly between 12 and 24 h of treatment.

**Figure 2:**
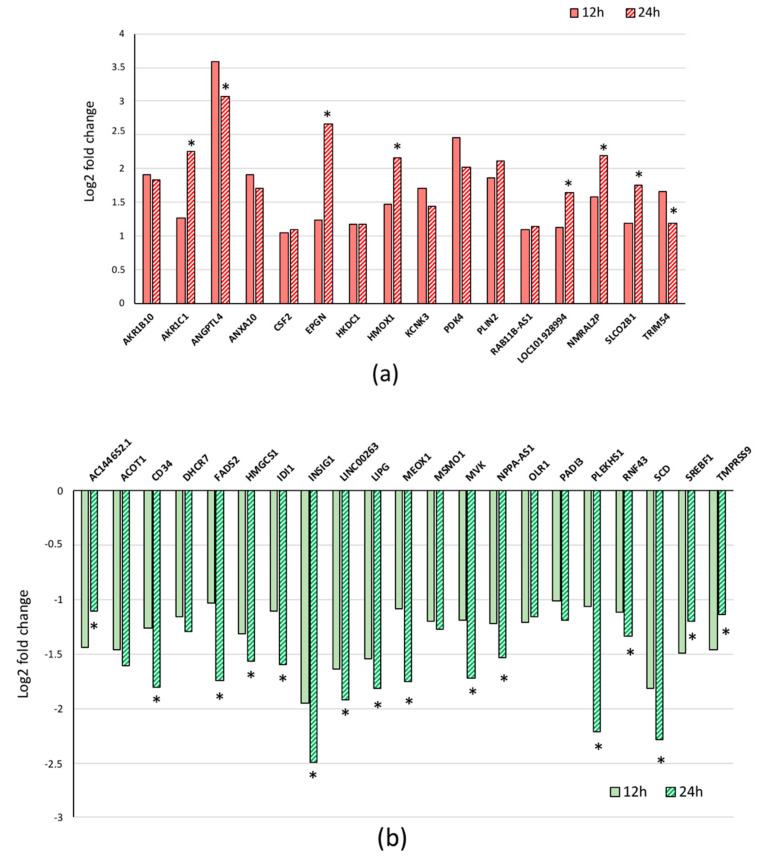
Differentially expressed genes with time-dependent effects of DHA. Log2 fold change of upregulated (**a**) and downregulated (**b**) genes at 12 h and 24 h after DHA treatment. Data were extracted from Table 1 and Table 2; the p values between DHA-treated samples and corresponding control samples were below 0.001 (Tables S2 and S3), and the comparison between treated samples at 12 h and 24 h was significantly different (*) with *p* < 0.001.

This obvious change in the pattern of gene expression in treated cells compared to control cells concerns a total of 390 genes differentially expressed in a statistically significant manner (i.e., Log2 fold change ≥1 and *p* < 0.001) after 24 h in the presence of DHA. A total number of 23 and 213 genes were upregulated in DHA-treated cells versus controls at 12 h and 24 h time points, respectively (Table 1 and Table S2). Among the 23 genes upregulated after 12 h of treatment, 16 were also differentially expressed at 24 h and only seven among them (i.e., *AKR1C1*, *EPGN*, *HMOX1*, *PLIN2*, *SLCO2B1*, and two LncRNA/pseudogene) are further upregulated showing a time-dependent effect of DHA (Figure 2a). By contrast, five genes (i.e., *AKR1C3*, *CPT1A*, *GCLM*, *HSPA6*, *SLC25A20*) and two LncRNA (*RP5-875H18.4* and *LINC01363*) among those upregulated at 12 h were not differentially expressed at 24 h.

Furthermore, 32 and 177 additional genes were downregulated in DHA-treated cells versus controls at 12 h and 24 h time points, respectively (Table 2 and Table S3). Among them, 17 genes were downregulated at 12 h and further decreased at 24 h (Figure 2b), showing a time-dependent effect of DHA. Besides, the expression level was less decreased at 24 h for three genes (i.e., *OLR1*, *SREBF1*, *TMPRSS9*) and one LncRNA.

**Figure 3:**
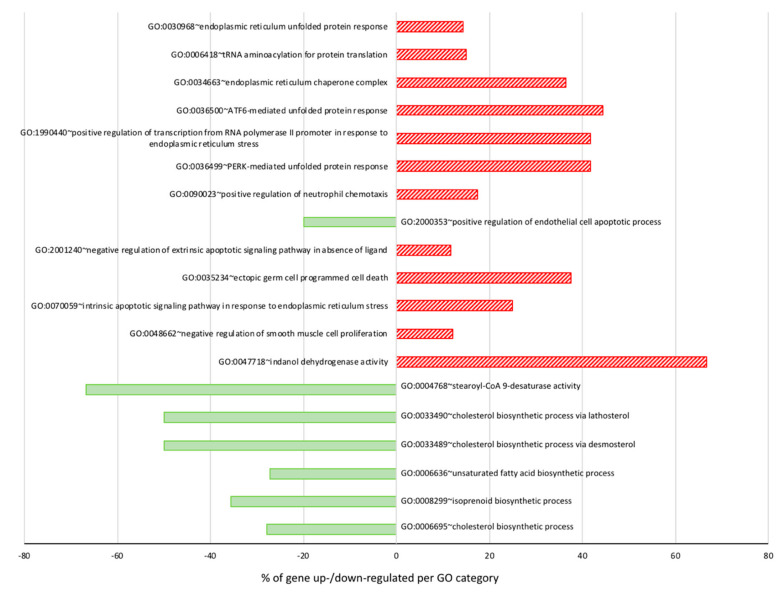
Percentage of genes per Gene Ontology category up- or downregulated following 24 h DHA treatment. Data were extracted from Table S4. Green bars on the left represent downregulated genes, whereas red hatched bars on the right are for upregulated group of genes.

These differentially expressed genes could be categorized in several Gene Ontology classes grouped into eight main groups (Table S4), including: (1) lipid and sterol metabolism, (2) cell growth/proliferation, (3) apoptosis, (4) cell adhesion, migration and invasion, (5) angiogenesis, (6) ER-stress response, (7) signaling pathways, and (8) miscellaneous. The main unidirectionally modulated pathways are represented in Figure 3, which show the percentage of genes per category that are up- or downregulated following 24 h of DHA treatment. The cholesterol biosynthesis pathway appears as clearly downregulated, and the ER-stress response pathway as obviously upregulated (Figure 3).

### 3.2. Quantitative RT-PCR Validation of the Results

**Figure 4:**
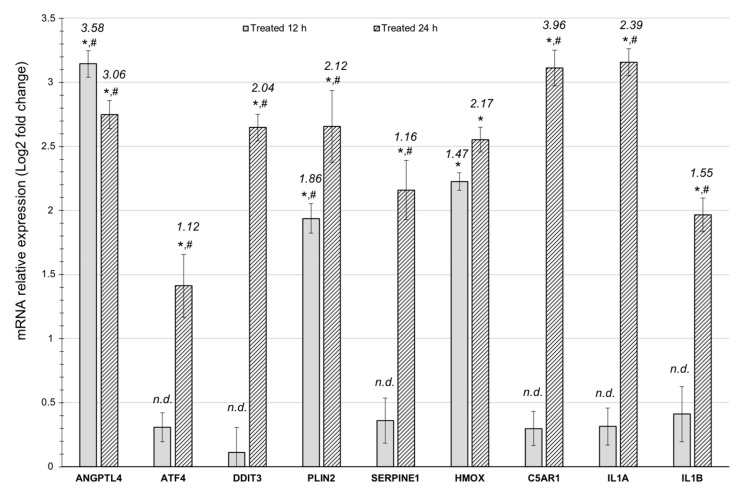
Quantitative RT-PCR validation of mRNA expression level. The results are expressed as the Log2 fold variation with respect to the corresponding control and *GAPDH* was used as a reference gene. Numeric values are the RNA-seq Log2 fold change values as per Supp. Table S2 for comparison; n.d. means that this gene was not differentially expressed in the RNA-seq dataset at the 12 h timepoint. Data are the mean +/- S.D. of three independent experiments; * means a significant difference with a corresponding control with *p* < 0.001 and # means significative difference between 24 h and 12 h treated timepoints with *p* < 0.001.

To validate the RNA-seq data, the expression level of nine genes was determined using quantitative RT-PCR (Figure 4). Even though the Log2 fold change varied to some extent between the RNA-seq data and the quantitative RT-PCR data due to technical differences and limitations, the expression trend of the tested genes was consistent between the two data sets. This indicates that the RNA-seq results are a reliable resource for further analysis.

### 3.3. DHA Impacted the Regulation of Lipids, Fatty Acid, and Sterol Metabolisms

Although the *CPT1A* (Carnitine Palmitoyltransferase 1A), the key enzyme of fatty acid degradation, gene was upregulated at 12 h post-DHA treatment, it was not differentially expressed thereafter. However, most of the genes (i.e., 11 out of 20) that were downregulated by DHA at both 12 h and 24 h are involved in fatty acid and cholesterol metabolisms and their regulation (Figure 2B, Table 2 and Tables S3 and S4). Ten additional genes involved in lipid or lipid-related metabolisms were also downregulated at 24 h, whereas seven genes of such pathways were upregulated. Thus, the number of genes related to lipid metabolism reaches 7% of the differentially expressed genes.

As it could be expected, the incorporation of DHA into the cells [23,37] resulted in the inhibition of the biosynthesis pathway of long-chain fatty acid as shown by the decreased expression of *Acyl-CoA thioesterase 1* (*ACOT1*), *Fatty Acid Elongase 3* (*ELOVL3*), *Stearoyl-CoA Desaturase* (*SCD*) and moreover long-chain polyunsaturated fatty acids (LCPUFAs) by decreasing the expression of the delta-6 desaturase *Fatty Acid Desaturase 2* (*FADS2*). Twenty-four hours of DHA treatment also decreased by 2.3-fold the expression of the *Fatty Acid Synthase* (*FASN*), and even if *Oleoyl-ACP Hydrolase* (*OLAH*) gene expression was four-fold increased. Besides, the *PNPLA3* gene, which encodes a triacylglycerol lipase, is two-fold decreased. Altogether, these data indicate that fatty acid metabolism and then energy metabolism may be strongly affected by DHA treatment, which may explain in part its antiproliferative effect. Besides, DHA was shown to inhibit the increase of lipogenic activity and gene expression (especially that of *FASN*) in *HER2*-overexpressing breast cancer cells, which was independent of the mTOR and PPARγ pathways [52], suggesting that DHA effect is also independent of these pathways.

**Figure 5:**
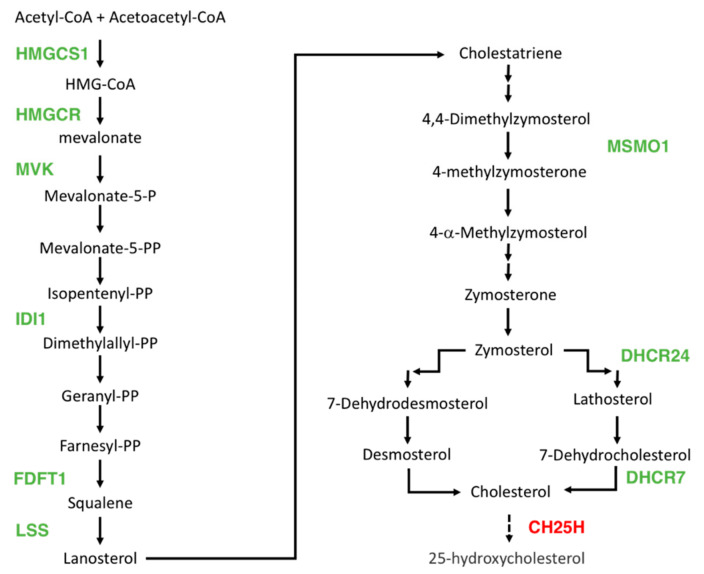
Schematic view of genes downregulated (green) and upregulated (red) in the biosynthesis and degradation pathway of cholesterol.

In addition, ten genes involved in cholesterol metabolism were differentially expressed after DHA treatment. Nine genes involved in the cholesterol biosynthesis pathway (i.e., *HMGCS1*, *HMGCR*, *MVK*, *IDI1*, *FDFT1*, *LSS*, *MSMO1*, *DHCR24*, and *DHCR7*) are downregulated (Figure 5), whereas one gene (*CH25H*) coding an enzyme involved in cholesterol catabolism was upregulated indicating that cholesterol may be dramatically affected by DHA treatment as previously observed [53,54].

In addition, some genes involved directly or indirectly in fatty acid and cholesterol metabolism regulation by the Sterol Regulatory Element Binding Transcription Factor 1 (SREBF1/SREBP1) or Peroxisome Proliferator-Activated Receptor alpha (PPAR-α) were downregulated. A three-fold decrease of *SREBF1* gene itself was observed at 12 h post-DHA treatment. This gene encodes a basic helix-loop-helix-leucine zipper (bHLH-Zip) transcription factor that binds to the sterol regulatory element-1 and then regulates transcription of the *LDL receptor* (*LDLR*) gene, as well as the fatty acid and to a lesser degree the cholesterol synthesis pathway. Indeed, the *LDLR* gene was also downregulated by about three-fold following DHA treatment. The *INSIG1* gene was dramatically downregulated by DHA both after 12 h (about four-fold) and 24 h (about five-fold) of treatment (Figure 2b). This gene encodes a transmembrane protein of the endoplasmic reticulum (ER) that regulates cholesterol metabolism, lipogenesis, and glucose homeostasis [55]. The INSIG1 protein binds the sterol-sensing domains of SREBP cleavage-activating protein (SCAP) and 3-hydroxy-3-methylglutaryl-coenzyme A reductase (HMG-CoA reductase), and is essential for the sterol-mediated trafficking of these two proteins [56]. In the same way, the *PDK4* gene, which encodes a pyruvate dehydrogenase kinase that plays a key role in the regulation of glucose and fatty acid metabolism and homeostasis via phosphorylation of the two pyruvate dehydrogenase subunits [57], is upregulated by 5.5- and four-fold at 12 h and 24 h, respectively (Figure 2a). By contrast, the *ANKRD1* gene is only early downregulated at 12 h but not at 24 h. The encoded protein may be a transcription factor involved in the regulation of lipid metabolism by PPAR-α [58].

The lipoprotein metabolism is also affected by the downregulation of the *LIPG* gene, which encodes a triglyceride lipase involved in high-density lipoproteins hydrolysis. The *Oxidized Low-Density Lipoprotein Receptor 1* (*OLR1*) gene expression is also reduced, at both time points (Figure 2b). However, the impact in cell cultures should rather be due to the role of OLR1 in the regulation of Fas-induced apoptosis [59].

By contrast, several genes involved in eicosanoid, lipophilic hormones, and vitamins are upregulated. This includes the aldo-keto reductases genes *AKR1B10*, *AKR1C1,* and *AKR1C3*, which are involved in retinoids, progesterone, and prostaglandin metabolisms, respectively. In addition, the *ALOX5AP* gene encoding a 5-lipoxygenase activating protein required for leukotriene synthesis is also upregulated, as well as the *PTGS2* gene encoding Prostaglandin-Endoperoxide Synthase 2 (or cyclooxygenase-2), the key enzyme of prostaglandin synthesis. This suggests that increasing PUFA content leads to the stimulation of storage and use paths. In agreement with this suggestion, the *Perilipin* (*PLIN2*) gene, which encodes a protein involved in the coating of intracellular lipid droplets [60,61], is strongly upregulated at both time points (Figure 2a). Finally, the *GPCPD1* gene that encodes Glycero-phosphocholine Phosphodiesterase 1 is also upregulated at 24 h (Tables S2 and S4).

It should be noted that the effect of DHA on breast cancer cells may be different in other cell lines due to their mesenchymal *versus* epithelial status. Indeed, the breast cancer epithelial cell model MCF7, with respect to the MDA-MB-231 mesenchymal model, displays increased lipogenic activity as highlighted by the higher level of FASN and other lipogenic enzymes whose expression is controlled transcriptionally [62]. Therefore, in further work, the transcriptomic response to DHA should also be investigated in MCF-7 cells.

### 3.4. Antiproliferative Effect and Induction of Apoptosis

Several genes involved in the control of cell growth and the proliferation, and cell death by apoptosis or autophagy were found as differentially expressed, as highlighted in Table S4. Many genes contribute to both cell growth and cell death pathways, as well as other biological processes. In addition, some of these gene products act in an opposite way depending on the cell type or condition. For example, *NUPR1* may act as a negative regulator of apoptosis as well as a facilitator of apoptosis [63,64] or *FGFR3*, which may promote apoptosis in chondrocytes, but can also promote cancer cell proliferation [65,66]. Therefore, it is not surprising to find these genes either down- or upregulated in the same category. However, some clear trends could be highlighted from Table S4.

Genes identified as negative regulators of cell proliferation such as *DLEC1*, which is the highest upregulated gene (about 30-fold increase), *KLF4*, *HMOX1*, *IL12A*, *NUPR1*, *TRIB1* are rather upregulate, whereas positive regulators of cell proliferation are downregulated such as *BMP4*, *CDKN2C*, *ERBB3*, *FGFR3*, *TGFB3*, and *WT1*. Interestingly, the *PLK1* gene is also downregulated in DHA-treated cells. It encodes a CDC5/Polo-Like Kinase that acts as a negative regulator of p53 family members and then as a critical regulator of cell cycle progression, mitosis, and cytokinesis [67,68]. The INSIG1 metabolic regulator, whose expression is downregulated in DHA-treated cells (see above), may also play a regulatory role during the G0/G1 transition of cell growth [69]. Genes that are involved in the mitotic process, such as *ASPM* and *SAPCD2* may also play a role in the positive regulation of cell proliferation, and tumor cell growth [70,71,72,73,74], the DHA-induced decrease of their expression is then consistent with the antiproliferative effect of DHA.

**Figure 6:**
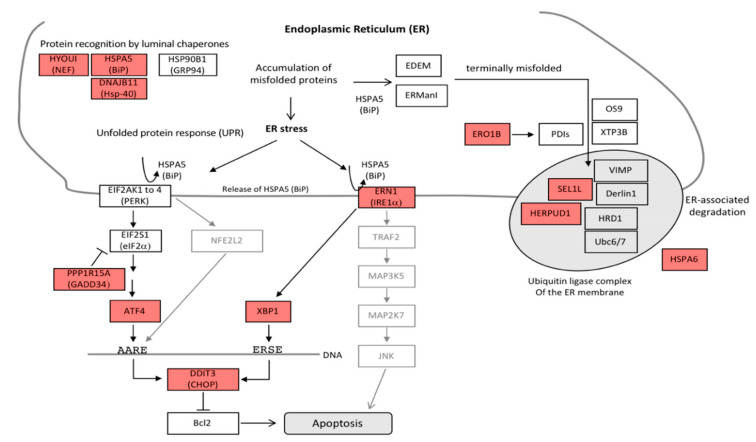
Schematic representation of upregulated genes (red boxes) related to ER-stress and leading to apoptosis and protein degradation. Common names of related proteins are indicated in brackets.

However, the most striking finding is the upregulation of 25% of the genes involved in the apoptotic signaling pathway in response to ER-stress, namely *ATF4*, *CEBPB* (or *NF-IL6*), *CHAC1*, *DDIT3* (also known as *CHOP*), *ERN1* (*IRE1-α*), *PPP1R15A* (*GADD34*), *TRIB3*, and *XBP1* (Table S4, Figure 3 and Figure 6) [75,76]. The upregulation of *INHBE* is also a marker of the ER-stress induction in DHA-treated cells and may act on cell growth inhibition [77]. The triggering of ER-stress-induced apoptosis by DHA agrees with previous studies using colon, colorectal, and glioma cancer cells [54,78,79,80] and even in non-alcoholic steatohepatitis patients [81]. In addition, the Glutathione-Specific Gamma-Glutamyl-cyclo-transferase encoded by the *CHAC1* gene, which is also upregulated, induces a glutathione depletion that is an important factor for apoptosis initiation and execution, and thus, acts as a pro-apoptotic component of the unfolded protein response pathway by mediating the pro-apoptotic effects of the ATF4-DDIT3/CHOP cascade [82]. Although it is always difficult to generalize results from one cancer lineage to another, previous studies in different cancer cell lines may provide some suggestions of the mechanisms by which DHA might act in MDA-MB-231 breast cancer cells to induce ER-stress and then apoptosis. In colon cancer cell lines, DHA enhanced lipid peroxidation followed by the increased levels of phosphorylated eIF2a, an early hallmark of ER-stress [54]. Prolonged ER-stress may lead to apoptosis through the activation of the ATF-4 transcription factor and DDIT3/CHOP [54], which agrees with our present results. In addition to being the principal site for protein synthesis and folding, ER is also the major site of Ca^++^ storage and signaling. In colon cancer cells, Jacobsen et al. have shown that DHA treatment mobilizes Ca^++^ from ER into the cytosol by an unknown mechanism, and then this Ca^++^ perturbation may promote ER-stress [54]. The perturbation of biosynthesis of fatty acids and cholesterol may also induce ER-stress. However, DHA reduced the cholesterol synthesis pathway in colon cancer cells [54], contrasting with our transcriptomic results in breast cancer cells (see above). The results of Shin et al., using the cisplatin-resistant gastric cancer cell line SNU601/cis2, suggest that increased ROS generation by DHA could induce ER stress via direct action on eIF2α kinases, working upstream of the inositol 1,4,5-triphosphate receptor and Ca^++^-mediated induction of the ER-stress response and apoptosis [80]. They also highlighted the involvement of the G-protein coupled receptor 120 (GPR120), a receptor of long-chain fatty acid [80], but this receptor does not appear to be expressed in MDA-MB-231 cells contrarily to MCF-7 breast cancer cells [83].

In addition, some other pro-apoptotic pathways may be stimulated by DHA through the up-regulation of *CEBPG*, *DDIT4*, *G0S2*, *PPP1R15A*, *UNC5B*, *PTPRH*, *SESN2*, and *TNFSF15* (Table 1 and Table S2). The CEBPG (CCAAT Enhancer Binding Protein Gamma) bZIP transcription factor may stimulate the Akt-dependent apoptotic pathway through its cooperation with FOS [84]. The G0/G1 Switch 2 (G0S2) protein promotes apoptosis by binding to BCL2, hence preventing the formation of protective BCL2-BAX heterodimers, and is related to the regulation of lipid metabolism by PPAR-α [85,86]. Besides, the *PPP1R15A* gene transcript levels are increased as previously reported following stressful growth arrest conditions and treatment with DNA-damaging agents [87]. The level of the PPP1R15A (Protein Phosphatase 1 Regulatory Subunit 15A) protein, also known as GADD34, is correlated with apoptosis through the recruitment of the serine/threonine-protein phosphatase PP1. Then, PPP1R15A downregulates the TGF-β signaling pathway by promoting dephosphorylation of TGFB1 by PP1 and apoptosis by inducing TP53 phosphorylation on Ser-15 [88,89]. The DDIT4 proteins also regulate p53/TP53-mediated apoptosis in response to DNA damage, but via its effect on mTORC1 activity [90]. The mTORC1 pathway may also be inhibited by Sestrin-2 (SESN2), which is known as a stress-inducible metabolic regulator stimulated, among other stresses, by ER-stress [91]. However, the *CASTOR3* and *Sestrin-3* (*SES3*) genes for mTORC1 regulators were found downregulated (Table 2 and Table S3). Finally, Sestrin-2, the expression of which is upregulated in DHA-treated cells, may also positively regulate the transcription by NFE2L2 of genes involved in response to oxidative stress by facilitating the SQSTM1-mediated autophagic degradation of KEAP1 [92]. The expression of the *SQSTM1* gene being also upregulated in DHA-treated cells (Table S2).

### 3.5. Reduction of Migration and Invasion

Migration and invasion of MBA-MD-231 cells were reduced by DHA-treatment as previously reported [37], but the mechanism of this still remains unknown. From the present RNA-seq analysis, no clear scheme could be drawn, and any specific pathway appears really modulated. However, several genes known, as involved in cell migration and invasion, are differentially expressed (Table S4). The protein encoded by the *TMPRSS9* gene is a membrane-bound type II serine polyprotease that is cleaved to release three different proteases. Two of the proteases are active and can be inhibited by serine protease inhibitors, and one is thought to be catalytically inactive. The *TMPRSS9* expression was reported to enhance the invasive capability of pancreatic cancer cells [93] and may be involved in cancer progression. Therefore, the two-fold downregulation of *TMPRSS9* may account, at least in part, for the reduction of the invasive phenotype of MDA-MB-231 cells. Similarly, the 2.5-fold downregulation of *ADAMTS4*, which encodes a protein of the “disintegrin and metalloproteinase with thrombospondin motifs” family responsible for the degradation of aggrecan, a major proteoglycan of cartilage, and brevican, a brain-specific extracellular matrix protein [94,95], may also account from some reduction of the invasive phenotype.

Maybe more relevant is the 2.3-fold downregulation of *ASAP3*, which encodes a member of a subfamily of ADP-ribosylation factor (Arf) GTPase-activating proteins. Indeed, the reduction of *ASAP3* expression levels slowed cell migration and invasion of hepatocellular carcinoma HepG2 and MDA-MB-231 breast cancer cells [96]. The 2.7-fold decrease of *CDC42BPG* gene expression, which is known as a key regulator of cell migration and cancer dissemination [97], is also a potential explanation of the reduction of MDA-MB-231 cell migration and invasion induced by DHA. The *CEMIP* (*KIAA1190*) gene was previously shown to play an important role in breast tumor growth and invasiveness [98,99], but, by contrast with previous genes, its early downregulation after 12 h of DHA-treatment was lost at 24 h (Table S3). The two-fold downregulation of the *CSTK* gene, encoding the Cathepsin-K lysosomal cysteine proteinase, which is initially involved in bone remodeling and resorption, is also of interest since Cathepsin-K is obviously involved in migration, invasion, and finally, bone metastasis of breast cancer cells [100,101].

In addition, the strong increase of *ANGPTL4* gene expression (12- and eight-fold at 12 h and 24 h, respectively—Table 1) can also be involved in the reduction of invasion. Indeed, *ANGPTL4* encodes a glycosylated, secreted protein containing a C-terminal fibrinogen domain, which is induced by peroxisome proliferation activators and functions as a serum hormone that mediates inactivation of the lipoprotein lipase LPL and, thereby, regulates glucose homeostasis, lipid metabolism, and insulin sensitivity [102]. However, this protein can also act as an apoptosis survival factor for vascular endothelial cells [103] and can prevent metastasis by inhibiting vascular growth and tumor cell invasion [103,104,105] although opposite effects can be observed under certain conditions or different cell types [106,107,108].

The *SERPINE1* gene is also upregulated. SERPINE-1 (previously known as PAI-1) is the principal inhibitor of tissue plasminogen activator (PLAT or tPA) and urokinase (PLAU or uPA), and hence, is an inhibitor of fibrinolysis, and also of breast cancer cell migration and invasion [109,110]. It is noteworthy that the *PLAT* gene is downregulated after 24 h of DHA treatment (Table S3). Altogether, the increase of *SERPINE-1* and the decrease of *PLAT* and *MMP11*, which act as SERPIN inhibitors [111], expression may lead to an important inhibitory effect on migration and invasion of DHA-treated cells, as previously observed [37].

Some other genes that are more or less directly involved in cell adhesion and migration are downregulated, such as *CD34*, *CLDN2*, *COL1A1*, *COL9A3*, *FAM20C*, and *NDNF* (Table S3). However, some inconsistencies should be noted such as the downregulation of the known negative regulator of breast cancer invasion or epithelial-mesenchymal transition *KLF17* [112,113,114] or the upregulation of extracellular matrix protein 2 (ECM2) gene and the collagen-induced receptor tyrosine kinase DDR2 gene, which is positively involved in breast tumor metastasis [115]. Similarly, the 2.4-fold upregulation of *calcireticulin* (*CALR*) and *S100P* genes, both of which encode calcium-binding proteins involved in the increased migration and invasion of breast cancer cells [116,117,118,119], is surprising and further research is needed to explain these apparent contradictions.

## 4. Conclusions

The transcriptomic analysis of DHA-treated MDA-MB-231 cells has confirmed that DHA incorporation leads to an important modification of gene expression profile, especially after 24 h of treatment. The impact of DHA-treatment is particularly visible concerning genes involved in the cholesterol biosynthesis pathway that is strongly downregulated and concerning the ER-stress response that is, conversely, upregulated. This transcriptomic induction of the ER-stress and unfolded protein response by DHA agreed with the results of a previous transcriptomic analysis of MDA-MB-231 cells showing a very similar pattern of differentially expressed genes after a short exposure time (6–8 h) to *Boswellia Serrata* ethanolic extract [120], which is rich in pentacyclic triterpenic acids but no DHA or eicosapentaenoic acid [121]. A second transcriptomic analysis of MDA-MB-231 cells after treatment with anacardic acid (24:1n5) for 6 h also showed the upregulation of ER-stress related genes, and highlighted the downregulation of *INSIG1* and *SCD* and upregulation of *PDK4* genes as observed in the present study [69]. Based on colon cancer studies [54], the perturbation of Ca^++^ storage and signaling, as well as lipid and cholesterol biosynthesis pathways by DHA are promoters of ER-stress. Besides the ER-stress and unfolded protein response, which could lead to the pro-apoptotic and antiproliferative effect of DHA, some other signaling pathways for cell growth and cell death are impacted such as the mTORC1, SMAD, Erk, and, to a lesser extent, Wnt pathways. Finally, DHA also impacted the expression of genes related to migration and invasion such as *CEMIP* (*KIAA1190*), as previously observed by Mazzio et al. [120], and especially the *SERPINE1*, *PLAT*, and *MMP11* trio. This transcriptomic analysis provides new evidence supporting the antiproliferative, pro-apoptotic, and anti-invasive effects of DHA. It will open new lines of investigation to understand its molecular mechanisms and support further research into the potential of DHA in the prevention of breast cancer.

## Figures and Tables

**Table 1:** Top 25 of DHA-upregulated genes (with the exception of pseudogenes and LncRNA).

Official Symbol *	Official Full Name (According to GeneCards^®^)	Main Functions (According to GeneCards^®^)	Log2 Fold Change	
			12h	24h
*DLEC1*	DLEC1 Cilia and Flagella Associated Protein	May act as a tumor suppressor by inhibiting cell proliferation		4.93
*ESRP1*	pithelial Splicing Regulatory Protein 1	mRNA splicing factor		4.12
*C5AR1*	Complement C5a Receptor 1	Signal transduction; immune response		3.96
*GDF15*	Growth Differentiation Factor 15	Signal transduction		3.95
*SHANK2*	SH3 And Multiple Ankyrin Repeat Domains 2	Synapse component		3.73
*MOV10L1*	Mov10 Like RISC Complex RNA Helicase 1	piRNA processing; transposable element inhibition		3.67
*INHBE*	Inhibin Subunit Beta E	Apoptosis inductor; proliferation inhibitor, ER stress response		3.43
*FLRT1*	Fibronectin Leucine Rich Transmembrane Protein 1	Signal transduction; cell adhesion		3.39
*ANGPTL4*	Angiopoietin Like 4	Lipoprotein metabolism; Regulation of lipid metabolism by PPARalpha	3.58	3.06
*SPX (C12orf39)*	Spexin Hormone	modulation of cardiovascular and renal function; energy metabolism and storage; inhibits adrenocortical cell proliferation		2.96
*KLHDC7B*	Kelch Domain Containing 7B	Unknown		2.91
*ASNS*	Asparagine Synthetase (Glutamine-Hydrolyzing)	Asparagin synthesis		2.81
*ECM2*	Extracellular Matrix Protein 2	Cell adhesion		2.78
*EPGN*	Epithelial Mitogen	Signal transduction; proliferation	1.23	2.67
*CTH*	Cystathionine Gamma-Lyase	Cysteine metabolism		2.59
*NUPR1*	Nuclear Protein 1, Transcriptional Regulator	Transcriptional regulator; stress response		2.48
*CLDN1*	Claudin 1	Tight junction component; water permeability		2.43
*IL1A*	Interleukin 1 Alpha	Signal transduction; inflammatory response		2.39
*SLC16A1*	Solute Carrier Family 16 Member 1	Monocarboxylate transporter		2.37
*HSPA5*	Heat Shock Protein Family A (Hsp70) Member 5	Endoplasmic reticulum chaperone; ER stress response		2.28
*AKR1C1*	Aldo-Keto Reductase Family 1 Member C1	Progesterone metabolism	1.26	2.25
*TRIB3*	Tribbles Pseudokinase 3	Signal transduction; inhibition of NF-KB pathway		2.22
*SLC6A9*	Solute Carrier Family 6 Member 9	Glycine transporter		2.18
*HMOX1*	Heme Oxygenase 1	Heme catabolism	1.47	2.17
*PLIN2*	Perilipin 2	Lipid droplets component; marker of lipid accumulation; Regulation of lipid metabolism by PPARalpha	1.86	2.12

* Gene symbol in brackets corresponds to former gene name.

**Table 2:** Top 25 of DHA-downregulated genes.

Official Symbol *	Official Full Name (According to GeneCards^®^)	Main Functions (According to GeneCards^®^)	Log2 Fold Change	
			12h	24h
*INSIG1*	Insulin Induced Gene 1	Regulation of cholesterol biosynthesis by SREBP (SREBF).	−1.95	−2.50
*SCD*	Stearoyl-CoA Desaturase	Fatty Acyl-CoA Biosynthesis; Regulation of cholesterol biosynthesis by SREBP (SREBF).	−1.82	−2.28
*PLEKHS1*	Pleckstrin Homology Domain Containing S1	Unknow	−1.07	−2.21
*SORBS2*	Sorbin And SH3 Domain Containing 2	Signal transduction; adaptator protein		−2.15
*SHISA3*	Shisa Family Member 3	Signal transduction; adaptator protein; Wnt pathway		−2.05
*LINC00263*	Long Intergenic Non-Protein Coding RNA 263	Unknow	−1.64	−1.92
*PDE4B*	Phosphodiesterase 4B	Signal transduction		−1.86
*NEURL1B*	Neuralized E3 Ubiquitin Protein Ligase 1B	E3 ubiquitin-protein ligase; Notch pathway		−1.82
*F13A1*	Coagulation Factor XIII A Chain	Clotting		−1.82
*LIPG*	Lipase G, Endothelial Type	Lipoprotein metabolism	−1.55	−1.82
*WT1*	Wilms’ Tumor-1 Transcription Factor	Transcription factor; cell proliferation; tumor suppressor		−1.81
*CD34*	CD-34 molecule	Surface antigen; possible adhesion molecule	−1.26	−1.80
*MEOX1*	Mesenchyme Homeobox 1	Transcription factor	−1.08	−1.76
*FADS2*	Fatty Acid Desaturase 2	Biosynthesis of highly unsaturated fatty acids	−1.03	−1.74
*MVK*	Mevalonate Kinase	Terpenoid backbone biosynthesis; Regulation of cholesterol biosynthesis by SREBP	−1.19	−1.72
*OLFML2B*	Olfactomedin Like 2B	Extracellular matrix binding		−1.63
*CAVIN2 (SDPR)*	Caveolae Associated Protein 2	Regulation of caveolae morphology		−1.62
*ACOT1*	Acyl-CoA Thioesterase 1	Fatty acid biosynthesis	−1.46	−1.61
*TENT5C (FAM46C)*	Terminal Nucleotidyltransferase 5C	mRNA stability; Mainly targets mRNAs encoding endoplasmic Reticulum-targeted protein; may be involved in the induction of cell death		−1.61
*RHOV*	Ras Homolog Family Member V	Signal transduction		−1.60
*IDI1*	Isopentenyl-Diphosphate Delta Isomerase 1	Terpenoid backbone biosynthesis; Regulation of cholesterol biosynthesis by SREBP	−1.11	−1.60
*ANO1*	Anoctamin 1	Calcium-activated chloride channel		−1.57
*HMGCS1*	3-Hydroxy-3-Methylglutaryl-CoA Synthase 1	Regulation of lipid metabolism by PPARalpha	−1.32	−1.56
*SESN3*	Sestrin 3	Protection against oxidative stress; negative regulation of mTOR pathway		−1.55
*FAM13C*	Family With Sequence Similarity 13 Member C	Unknow		−1.55

* Gene symbol in brackets corresponds to former gene name.

## Supplementary Materials

The following are available online at https://www.mdpi.com/1660-4601/17/10/3746/s1, Table S1: Primer sequence used for quantitative RT-PCR analysis, Table S2: Alphabetical list and Log2 fold change values of significantly DHA-upregulated genes, including protein-coding genes, pseudogenes, and RNA coding genes, Table S3: Alphabetical list and Log2 fold change values of significantly DHA-downregulated genes, including protein-coding genes, pseudogenes, and RNA coding genes, Table S4: Gene Ontology classification of upregulated (red) and downregulated (green) genes after 24h of DHA treatment.
